# CdrA Interactions within the Pseudomonas aeruginosa Biofilm Matrix Safeguard It from Proteolysis and Promote Cellular Packing

**DOI:** 10.1128/mBio.01376-18

**Published:** 2018-09-25

**Authors:** Courtney Reichhardt, Cynthis Wong, Daniel Passos da Silva, Daniel J. Wozniak, Matthew R. Parsek

**Affiliations:** a Department of Microbiology, University of Washington, Seattle, Washington, USA; b Departments of Microbial Infection and Immunity, Microbiology, The Ohio State University, Columbus, Ohio, USA; Emory University School of Medicine

**Keywords:** CdrA, *Pseudomonas aeruginosa*, Psl, biofilm, elastase, exopolysaccharides

## Abstract

Biofilms are robust multicellular aggregates of bacteria that are encased in an extracellular matrix. Different bacterial species have been shown to use a range of biopolymers to build their matrices. Pseudomonas aeruginosa is a model organism for the laboratory study of biofilms, and past work has suggested that exopolysaccharides are a required matrix component. However, we found that expression of the matrix protein CdrA, in the absence of biofilm exopolysaccharides, allowed biofilm formation through the production of a CdrA-rich proteinaceous matrix. This represents a novel function for CdrA. Similar observations have been made for other species such as Escherichia coli and Staphylococcus aureus, which can utilize protein-dominant biofilm matrices. However, we found that these CdrA-containing matrices were susceptible to both exogenous and self-produced proteases. We previously reported that CdrA directly binds the biofilm matrix exopolysaccharide Psl. Now we have found that when CdrA bound to Psl, it was protected from proteolysis. Together, these results support the idea of the importance of multibiomolecular components in matrix stability and led us to propose a model in which CdrA-CdrA interactions can enhance cell-cell packing in an aggregate that is resistant to physical shear, while Psl-CdrA interactions enhance aggregate integrity in the presence of self-produced and exogenous proteases.

## Abstract (precis)

Pseudomonas aeruginosa forms multicellular aggregates or biofilms using both exopolysaccharides and the CdrA matrix adhesin. We showed for the first time that P. aeruginosa can use CdrA to build biofilms that do not require known matrix exopolysaccharides. It is appreciated that biofilm growth is protective against environmental assaults. However, little is known about how the interactions between individual matrix components aid in this protection. We found that interactions between CdrA and the exopolysaccharide Psl fortify the matrix by preventing CdrA proteolysis. When both components—CdrA and Psl—are part of the matrix, robust aggregates form that are tightly packed and protease resistant. These findings provide insight into how biofilms persist in protease-rich host environments.

## INTRODUCTION

Most microbes can form multicellular communities called biofilms that are encased in an extracellular matrix that is typically rich in polymeric biomolecules such as polysaccharides, proteins, and DNA (1–6). Biofilm matrix compositions differ across species and growth conditions. However, in general, the matrix serves as both a structural scaffold and a protective shield against external assaults such as antibiotic treatment or host defenses (6–11). Pseudomonas aeruginosa is a model organism for studying biofilms in the laboratory and also causes chronic infections (12–16). The impact of exopolysaccharides (EPS) on P. aeruginosa biofilm communities has been fairly well studied (17). However, the different roles that proteins may play in the biofilm matrix are less clear (18).

**FIG 1:**
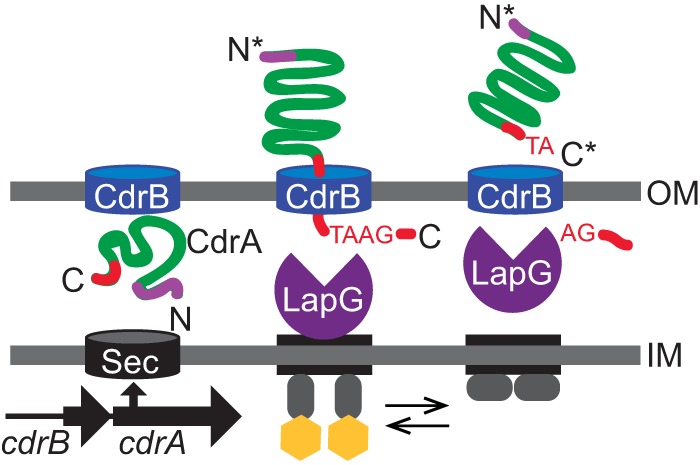
CdrA is the cargo of the two-partner secretion system encoded by the *cdrAB* operon. CdrA is found in both cell-associated and secreted fractions. Periplasmic protease LapG can cleave CdrA near its C terminus, which liberates CdrA from the bacterial cell surface. (Adapted from reference 20 with permission of the publisher.)

The first biofilm matrix protein to be identified in P. aeruginosa was CdrA (19), which serves as the cargo of the two-partner secretion (TPS) system encoded by the *cdrAB* operon. The outer membrane pore, CdrB, is necessary for export of CdrA from the periplasm to the cell-surface. CdrA is predicted to be structurally similar to other TPS proteins such as filamentous hemagglutinin (FHA), including a β-helical motif that makes up the elongated fibrillar protein core (19). CdrA is present in both cell-associated and supernatant fractions (19). The *cdrA* gene encodes a 220-kDa protein, and yet free CdrA that is released away from the cellular surface is only 150 kDa in size and is truncated such that it primarily contains only the predicted fibrillar core. Cleavage at the CdrA N terminus occurs nearly 400 residues after the predicted Sec signal. The mechanism of this cleavage is unknown. Recent findings demonstrated that cleavage at the CdrA C terminus occurs via LapG, which is a periplasmic protease that is regulated by the intracellular signaling molecule cyclic di-GMP (c-di-GMP). Cleavage by LapG results in release of CdrA from the cellular surface under conditions of low c-di-GMP levels (20, 21). The processing of CdrA is depicted in the diagram in Fig. 1.

In addition to the adhesin CdrA (19), matrix components of nonmucoid P. aeruginosa biofilms include the EPS Psl and Pel (17, 22). CdrA, Psl, and Pel are each c-di-GMP dependent (19, 22–26). The CdrA structure is predicted to contain sugar binding and carbohydrate-dependent hemagglutination domains that may be important for its interactions with matrix EPS and/or host molecules. The structural stability that CdrA lends to the biofilm is hypothesized to be partly due to Psl binding. Psl consists of a repeating pentasaccharide consisting of mannose, rhamnose, and glucose in a 3:1:1 ratio (19, 27–29). The composition of Psl is distinct from that of either alginate or Pel (30, 31). Evidence of the specific interaction between CdrA and Psl includes findings showing that CdrA and Psl coimmunoprecipitate (Co-IP) from liquid culture supernatant and that CdrA promotes Psl-dependent aggregation in liquid culture (19).

Several CdrA homologs in other species can mediate bacterial aggregation independently of polysaccharides. These include the adhesins FHA (32), antigen 43 (Ag43) (33), and AIDA (34). Thus, we sought to determine if CdrA could act to tether together bacteria independently of Psl (35). We demonstrated that CdrA can mediate bacterial aggregation and biofilm adherence even in the absence of Psl or other biofilm EPS. We provide evidence that this is likely due to CdrA-CdrA interactions.

The biofilm lifestyle is important to bacterial persistence, and so it is not surprising that P. aeruginosa has multiple, potentially redundant mechanisms for assembly of biofilms. However, we hypothesized that a CdrA/protein-dominant biofilm matrix would be sensitive to proteolytic degradation. This was found to be the case. Possession of such a proteolytically labile matrix could be detrimental to biofilm aggregate stability as P. aeruginosa produces its own slew of extracellular proteases (36, 37) and also is found in environments that are rich in exogenous proteases (38). Interestingly, we found that the P. aeruginosa EPS Psl protects CdrA from proteolytic cleavage. Additionally, we determined that the self-produced protease elastase (LasB) degrades CdrA. Thus, we envision that Psl-CdrA interactions can contribute to biofilm integrity and that they suggest an advantage for utilizing both proteins and EPS in the matrix. Collectively, our data support a model where CdrA promotes tight cellular interactions in biofilm aggregates, while Psl-CdrA interactions protect the matrix protein from proteolytic cleavage.

## RESULTS

### CdrA can mediate bacterial aggregation and static biofilm formation independently of known EPS.

We hypothesized that CdrA could mediate bacterial aggregation in the absence of Psl or other EPS. To test this hypothesis, the relative percentages of aggregation of strain PAO1 Δ*cdrA* and mutant strains that no longer produced Psl and/or other EPS (Pel and alginate) were evaluated after induction of *cdrAB* with arabinose. We also examined CdrA-dependent aggregation in the PAO1 Δ*wspF* strain background because it has been shown to have higher levels of Psl and CdrA.

**FIG 2:**
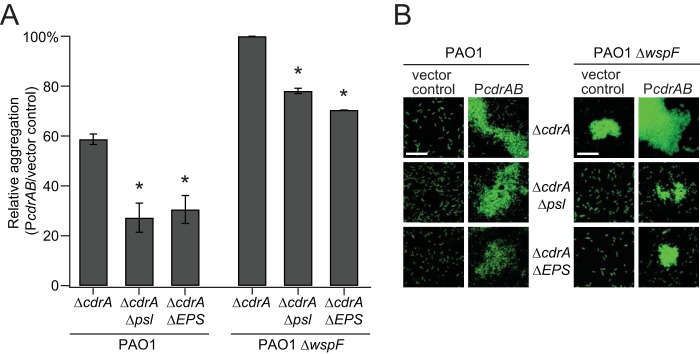
CdrA can mediate bacterial aggregation in the absence of Psl or other EPS. (A) Aggregation of wild-type PAO1 and mutant strains that no longer produce Psl and/or other EPS (Pel and alginate) was evaluated after induction of *PcdrAB* with arabinose. Relative aggregation levels were determined by calculating the difference in OD_600_ between the P*cdrAB* strain and its corresponding vector control strain, dividing by the OD_600_ of the vector control strain, and then multiplying by 100%. Data represent the means of results from three replicates, and error bars indicate standard deviations. An asterisk indicates a significant difference in the levels of aggregation of Psl and EPS mutants compared to their parent strains (either strain PAO1 Δ*cdrA* P*cdrAB* or strain PAO1 Δ*wspF* Δ*cdrA* P*cdrAB*) (Student’s *t* test; *P* < 0.05). (B) Aggregates of bacteria constitutively expressing GFP were imaged using confocal laser scanning microscopy. Representative images of each strain are shown and were obtained from microscopy of at least three biological replicates. Scale bars represent 25 μm, and “Δ*psl pel algD*” is abbreviated as “Δ*EPS*.”

Aggregation results in a decrease in the optical density at 600 nm (OD_600_) of the culture. Therefore, percent aggregation was determined by comparison of the OD_600_ of the P*cdrAB* strains to that of their isogenic empty vector control strains. Aggregates formed in all cases where *cdrAB* was induced (Fig. 2A; see also Fig. S1 in the supplemental material). Levels of aggregation were higher for strains producing EPS (*P* < 0.05) and were also higher in the PAO1 Δ*wspF* strain background than in the PAO1 background (*P* < 0.005).

10.1128/mBio.01376-18.1 FIG S1 Aggregation of wild-type PAO1 and mutant strains that no longer produce Psl and/or other EPS (Pel and alginate) was evaluated after induction of *PcdrAB* with arabinose. The OD_600_ values used to calculate the amount of aggregation are shown in the plot. Data represent the means of results from three replicates, and error bars indicate standard deviations. Download FIG S1, EPS file, 0.77 MB. Copyright © 2018 Reichhardt et al. 2018 Reichhardt et al. This content is distributed under the terms of the Creative Commons Attribution 4.0 International license.

Additionally, we observed bacterial aggregation using microscopy. For this experiment, we used bacteria that constitutively expressed green fluorescent protein (GFP). Again, bacteria aggregated when *cdrAB* was overexpressed, and this aggregation was most pronounced when the EPS Psl was also produced (Fig. 2B). Strains transformed with the vector control did not form aggregates. An exception was strain PAO1 Δ*wspF* Δ*cdrA*, which, due to its high level of EPS expression, formed small aggregates even without *cdrAB* overexpression. However, these EPS-only aggregates, unlike CdrA-mediated aggregates, were susceptible to disruption with a vortex mixer (Fig. S2).

10.1128/mBio.01376-18.2 FIG S2 Results of microscopy of aggregates formed by strains PAO1 Δ*wspF* Δ*cdrA* P*cdrAB* (protein plus EPS) and PAO1 Δ*wspF* Δ*EPS* Δ*cdrA* P*cdrAB* (protein only) and a PAO1 Δ*wspF* Δ*cdrA* vector control (EPS only) are shown. The aggregates were subjected to disruption with a vortex mixer (15 s). The EPS-only aggregates, unlike the CdrA-mediated aggregates, were susceptible to disruption with a vortex mixer. Download FIG S2, EPS file, 1.32 MB. Copyright © 2018 Reichhardt et al. 2018 Reichhardt et al. This content is distributed under the terms of the Creative Commons Attribution 4.0 International license.

**FIG 3:**
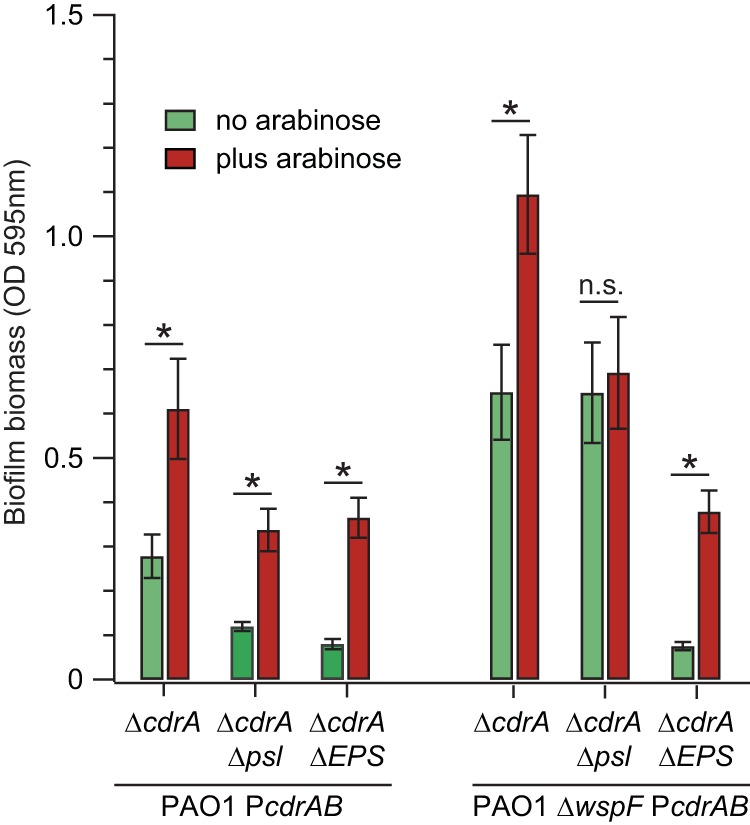
CdrA can mediate static biofilm formation in the absence of Psl or EPS. Static biofilm formation of *cdrAB* overexpression strains was measured by crystal violet staining. Green bars indicate control treatments without arabinose induction, and red bars indicate arabinose induction treatment. Data represent the means of results from six replicates, and error bars indicate standard deviations. An asterisk indicates a significant difference in biofilm biomass compared to the uninduced control (Student’s *t* test; *P* < 0.0005); n.s., not statistically significant compared to the uninduced control. “Δ*psl pel algD*” is abbreviated as “Δ*EPS*.”

Next, we sought to determine if CdrA could mediate static biofilm formation in the absence of Psl or other EPS. Static biofilm formation of *cdrAB* overexpression strains and their isogenic vector control strains was measured by crystal violet staining. In Fig. 3, the amount of adherent biofilm biomass is shown for both uninduced and arabinose-induced *cdrAB* expression. Similarly to the aggregation assay, we observed that induction of *cdrAB* expression resulted in increased static biofilm formation. In general, this CdrA-dependent increase in static biofilm formation occurred regardless of the presence or absence of EPS production. Also, more biofilm biomass was observed for strains PAO1 Δ*wspF* Δ*cdrA* and PAO1 Δ*wspF* Δ*cdrA* Δ*psl* due to an increase in Psl and Pel levels from the Δ*wspF* mutation. It should be noted that for strain PAO1 Δ*wspF* Δ*cdrA* Δ*psl*, overexpression of *cdrAB* did not result in an increase in static biofilm formation relative to its uninduced control. We repeated this assay, and again we observed that overexpression of *cdrAB* did not result in increased static biofilm formation for strain PAO1 Δ*wspF* Δ*cdrA* Δ*psl*. We are unsure of the reason for this result, as overexpression of *cdrAB* results in increased biofilm formation for strain PAO1 Δ*cdrA* Δ*psl* and both total EPS mutants PAO1 Δ*cdrA* Δ*EPS* and PAO1 Δ*wspF* Δ*cdrA* Δ*EPS*. In all cases, biofilm formation by strains transformed with only the vector control was similar to that observed for the isogenic uninduced P*cdrAB* strains (Fig. S3).

10.1128/mBio.01376-18.3 FIG S3 CdrA can mediate static biofilm formation in the absence of Psl or EPS. Static biofilm formation of *cdrAB* overexpression strains was measured by crystal violet staining. Green bars indicate control treatments without arabinose induction, and red bars indicate arabinose induction treatment. In all cases, the biofilm formation of strains transformed with only the vector control (vc) was similar to the biofilm formation seen with isogenic uninduced P*cdrAB* strains. Data represent the means of results from six replicates, and error bars indicate standard deviations. Download FIG S3, EPS file, 0.90 MB. Copyright © 2018 Reichhardt et al. 2018 Reichhardt et al. This content is distributed under the terms of the Creative Commons Attribution 4.0 International license.

### CdrA-CdrA interactions promote bacterial aggregation in the absence of EPS.

**FIG 4:**
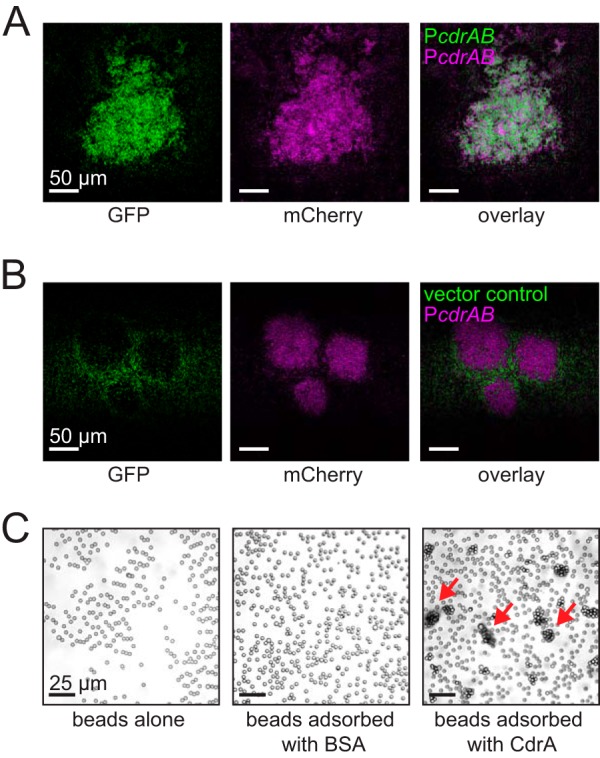
CdrA-CdrA interactions are likely responsible for EPS-independent aggregation. **(**A and B) Microscopy of aggregates formed by strain PAO1 Δ*wspF* Δ*cdrA* Δ*EPS* transformed with either P*cdrAB* or the empty vector control. For this experiment, mixed-culture aggregates were grown from a 1:1 inoculum of each strain. (A) The mixed culture of P*cdrAB* (GFP^+^) and P*cdrAB* (mCherry^+^) showed intermixing of the two strains. (B) The mixed culture of the empty vector control (GFP^+^) and P*cdrAB* (mCherry^+^) did not show mixing of the two strains. Representative images of each condition are shown and were obtained from microscopy of at least three biological replicates. (C) Light microscopy showed that beads aggregated when they were adsorbed with CdrA. Beads adsorbed with BSA or treated only with PBS buffer did not aggregate. Red arrows indicate some of the aggregates that were observed when beads were adsorbed with CdrA. The experiment was repeated three times, and representative images for each condition are shown.

Possible mechanisms of EPS-independent CdrA-mediated aggregation include (i) intercellular CdrA-CdrA interactions and (ii) an intercellular interaction between CdrA and another bacterial surface component(s) or both. To distinguish between these possibilities, the arabinose-inducible overexpression vector P*cdrAB* and the empty vector control were transformed into strain PAO1 Δ*wspF* Δ*cdrA* Δ*EPS* constitutively expressing either the fluorescent protein GFP or mCherry. Mixed cultures were grown with arabinose to induce expression and imaged using confocal laser scanning microscopy. As shown in Fig. 4A, the mixed culture of strain PAO1 Δ*wspF* Δ*cdrA* Δ*EPS* P*cdrAB* (GFP positive [GFP^+^]) and strain PAO1 Δ*wspF* Δ*cdrA* Δ*EPS* P*cdrAB* (mCherry^+^) formed coaggregates. In contrast, the mixed culture of strain PAO1 Δ*wspF* Δ*cdrA* Δ*EPS* P*cdrAB* (mCherry^+^) and strain PAO1 Δ*wspF* Δ*cdrA* Δ*EPS*-empty vector control (GFP^+^) formed aggregates that contained only the CdrA-positive bacteria (mCherry^+^) (Fig. 4B). Bacteria with only the vector control were excluded from the aggregates. These results support the idea that CdrA-CdrA interactions facilitate bacterial aggregation in the absence of EPS.

To further explore if CdrA-CdrA interactions can cause aggregation, we passively adsorbed purified CdrA to the surface of 3-μm-diameter latex beads and used light microscopy to observe the beads for aggregation. A schematic of this protocol is shown in Fig. S4. Beads aggregated when CdrA was adsorbed and did not aggregate when the beads were either incubated with phosphate-buffered saline (PBS) buffer alone or adsorbed with bovine serum albumin (BSA) (Fig. 4C). This result shows that CdrA is sufficient for aggregation and does not require any other P. aeruginosa surface molecule(s).

10.1128/mBio.01376-18.4 FIG S4 The schematic outlines the method used to determine if CdrA-CdrA interactions can cause aggregation. For this assay, purified CdrA was passively adsorbed to the surface of 3-μm-diameter latex beads and light microscopy was used to observe the beads for aggregation. When CdrA-CdrA interactions occurred, the beads aggregated. This is the result that was observed experimentally. Download FIG S4, EPS file, 0.64 MB. Copyright © 2018 Reichhardt et al. 2018 Reichhardt et al. This content is distributed under the terms of the Creative Commons Attribution 4.0 International license.

### CdrA-only biofilms are susceptible to proteases.

**FIG 5:**
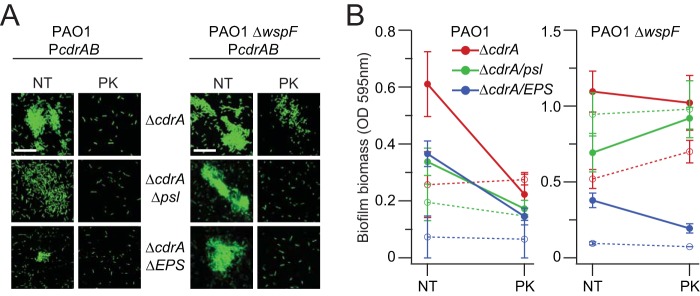
Proteinase K diminishes the amount of CdrA-dependent aggregation and static biofilm formation. (A) Aggregates of bacteria constitutively expressing GFP were imaged using confocal laser scanning microscopy with proteinase K (PK) treatment or no treatment (NT). Representative images of each strain and condition are shown and were obtained from microscopy of at least three biological replicates. (B) Static biofilm formation of *cdrAB* overexpression strains (solid lines) and isogenic strains carrying the empty vector control (dashed lines) was measured by crystal violet staining with PK treatment or NT. Data represent the means of results from 3 to 6 replicates, and error bars indicate standard deviations. Scale bars represent 25 μm, and “Δ*psl pel algD*” is abbreviated as “Δ*EPS*.”

We hypothesized that Psl may protect CdrA from proteolysis. To test this hypothesis, we treated CdrA-dependent bacterial aggregates with proteinase K (PK) and monitored the subsequent aggregation state using microscopy. PK has broad specificity and so was useful as an initial screen of CdrA proteolytic susceptibility. As shown in Fig. 5A, we observed disaggregation following PK treatment although this was not completely eliminated in strain PAO1 Δ*wspF* Δ*cdrA* P*cdrAB*. PK indeed proteolyzed CdrA as revealed by Western blot analysis of treated and untreated CdrA samples, and PK did not reduce bacterial viability under these assay conditions (Fig. S5).

10.1128/mBio.01376-18.5 FIG S5 Proteinase K was found to have no significant effect on the viability of cells. (A) Western blot analysis was used to demonstrate that proteinase K (PK) completely proteolyzed CdrA. (B) Four different P. aeruginosa strains were tested for viability before and after proteinase K treatment. CFU levels were determined following incubation with proteinase K. Incubation was 30 min at room temperature with rocking. Data represent the means of results from three biological replicates, and error bars indicate standard deviations. Download FIG S5, EPS file, 1.01 MB. Copyright © 2018 Reichhardt et al. 2018 Reichhardt et al. This content is distributed under the terms of the Creative Commons Attribution 4.0 International license.

Next, we treated preformed static biofilms of *cdrAB* overexpression strains with PK. As shown in Fig. 5B, we observed that PK treatment reduced the amount of CdrA-dependent static biofilm formation for all PAO1 Δ*cdrA* P*cdrAB* strains (solid lines) (*P* < 0.005) and not for the isogenic vector controls (dashed lines) (*P  > *0.05). In contrast, for the PAO1 Δ*wspF* Δ*cdrA* strains, PK treatment reduced the amount of biofilm biomass only for the Δ*EPS* strain (*P* < 0.005). This result suggested that EPS may protect CdrA from proteolytic degradation. That EPS is protective in the strain PAO1 Δ*wspF* background and not for PAO1 is predicted to be due to the higher levels of Psl and Pel that are made by strain PAO1 Δ*wspF*.

### Psl protects CdrA from endogenous proteases.

**FIG 6:**
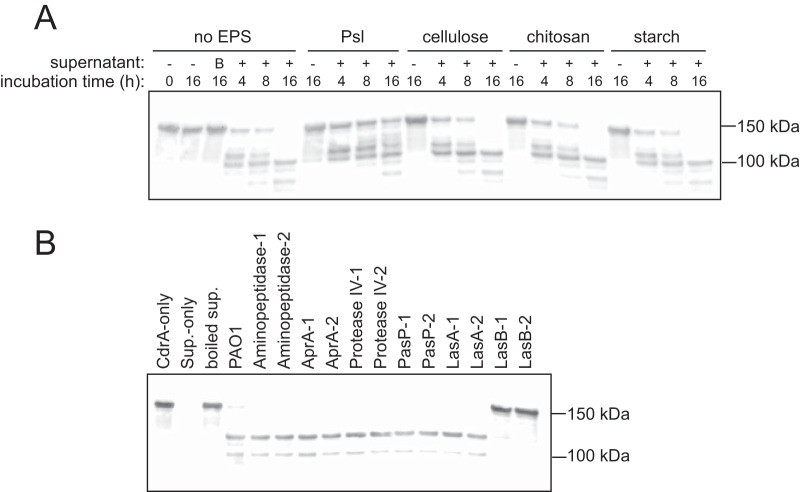
CdrA is susceptible to P. aeruginosa proteases, and CdrA-Psl interactions are protective. (A) Anti-CdrA Western blot analysis showed that Psl, but not cellulose, chitosan, or starch, protected CdrA from degradation by P. aeruginosa supernatant proteases. Intact secreted CdrA that had not been treated with supernatant was detected at 150 kDa. Treating CdrA with boiled supernatant (indicated as “B” above lane 3) did not result in CdrA proteolysis. (B) Anti-CdrA Western blot analysis showed that LasB proteolyzed CdrA. Purified CdrA was treated with cell-free stationary-phase supernatant collected from a panel of protease mutants from the P. aeruginosa PAO1 transposon mutant library. Six extracellular proteases (aminopeptidase, AprA, protease IV, PasP, LasA, and LasB) were surveyed, and two mutants were tested for each protease.

P. aeruginosa makes several secreted proteases. To test whether these self-produced proteases were able to degrade CdrA, we incubated purified CdrA with stationary-phase culture supernatant from strain PAO1 Δ*wspF* Δ*cdrA* Δ*EPS.* As shown in the Western blot analysis in Fig. 6A, we observed that CdrA (molecular weight [MW], 150 kDa) was degraded to lower-molecular-weight fragments following incubation with culture supernatants. Proteolysis was not observed when CdrA was incubated with boiled supernatant preparations. As the incubation time increased from 4 h to 16 h, CdrA was proteolyzed to fragments that were increasingly lower in molecular weight. By quantifying the intensity of the Western blot band at 150 kDa, we found that by 16 h, more than 90% of the starting CdrA had been proteolyzed. These results support the idea that CdrA is susceptible to endogenous proteases. On the basis of the possible protection of CdrA by Psl in the PK-treated static biofilm assay, we hypothesized that Psl may protect CdrA from degradation by endogenous proteases. To test this hypothesis, we incubated purified CdrA with isolated Psl as well as with the commercially available polysaccharides cellulose, chitosan, and starch, prior to treatment with culture supernatant. As shown in Fig. 6A, we observed that preincubation of CdrA with Psl, but not with cellulose, chitosan, or starch, protected CdrA from proteolysis. In fact, by 16 h of incubation with supernatant preparations, only 45% of the starting CdrA was proteolyzed when CdrA was preincubated with Psl. In contrast, incubation with the other polysaccharides did not provide protection, and approximately 90% of the starting CdrA was degraded, similarly to what was observed when the CdrA-only preparation was treated with supernatant preparations (see Fig. S6).

10.1128/mBio.01376-18.6 FIG S6 To determine the amount of CdrA that was degraded under the various incubation conditions, the intensity of the 150-kDa band in the Western blot was quantified. This band corresponds to intact, secreted CdrA. The plot shows the resulting arbitrary intensity units of the 150-kDa band. The quantification was performed using ImageJ software. Download FIG S6, EPS file, 0.71 MB. Copyright © 2018 Reichhardt et al. 2018 Reichhardt et al. This content is distributed under the terms of the Creative Commons Attribution 4.0 International license.

To determine if a specific self-produced P. aeruginosa protease was responsible for CdrA degradation, we tested a panel of protease mutants from the P. aeruginosa PAO1 transposon mutant library (39). Six extracellular proteases (aminopeptidase, AprA, protease IV, PasP, LasA, and LasB) were surveyed, and two mutants were tested for each protease. These proteases were chosen because they were identified in a proteomic screen of P. aeruginosa biofilm matrix-associated proteins (36). Purified CdrA was incubated with cell-free supernatants collected from stationary-phase cultures of each strain as well as the PAO1 isogenic background strain. Proteolysis was monitored by Western blot analysis of CdrA. Only P. aeruginosa mutants lacking *lasB* failed to cleave CdrA (Fig. 6B). The proteolytic activity of each strain was verified further using a zymogram gel with gelatin and casein (Fig. S7). This result suggests that the P. aeruginosa elastase LasB proteolyzes CdrA.

10.1128/mBio.01376-18.7 FIG S7 Protease activity in each of the supernatants was tested by using a zymogram gel with gelatin and casein. Zymogram gels represent a method of protein gel electrophoresis in which proteins are run under nondenaturing conditions. The gel matrix contains the protease substrates gelatin and casein. The proteases then “clear” a region of the gel via their enzymatic activity. The cleared bands correspond to the molecular weight of the nondenatured enzyme. Protease activity was detected in two regions of the gel, near 150 kDa and 50 kDa. Download FIG S7, EPS file, 0.76 MB. Copyright © 2018 Reichhardt et al. 2018 Reichhardt et al. This content is distributed under the terms of the Creative Commons Attribution 4.0 International license.

The observed protection of CdrA from proteolytic cleavage could be due to either an interaction between CdrA and Psl or an interaction between the protease and Psl. To distinguish between these possibilities, we tested the protease susceptibility of BSA following preincubation with Psl. If the protection was due to an interaction between the protease and Psl, we would expect that BSA would be protected from proteolysis. Instead, BSA was proteolyzed upon incubation with culture supernatants despite preincubation with Psl (Fig. S8). This result supports the idea that the protection provided to CdrA by Psl is likely due to an interaction between CdrA and Psl.

10.1128/mBio.01376-18.8 FIG S8 Bovine serum albumin (BSA) was treated with cell-free culture stationary-phase supernatants. SDS-PAGE analysis was performed with Coomassie staining, and the results showed that BSA was susceptible to proteolysis. Preincubation of BSA with Psl did not prevent proteolysis of BSA by supernatant proteases. Download FIG S8, EPS file, 0.58 MB. Copyright © 2018 Reichhardt et al. 2018 Reichhardt et al. This content is distributed under the terms of the Creative Commons Attribution 4.0 International license.

## DISCUSSION

We found that the P. aeruginosa biofilm adhesin CdrA is able to promote bacterial aggregation and biofilm formation independently of EPS. This represents a novel mechanism of action of CdrA. Protein-only or protein-dominant biofilm matrices have been described in other systems, including Staphylococcus aureus (40) and Escherichia coli (41, 42). This report provides new evidence that P. aeruginosa is similarly capable of forming biofilms without known EPS, although which environments or conditions favor these biofilms is unclear. While it is possible that CdrA could be interacting with a yet-to-be-discovered EPS, we believe that this is unlikely since extensive work performed in studying P. aeruginosa, including genomic sequencing, has not uncovered additional EPS candidates. As seen in other systems (33, 34), and as shown now for P. aeruginosa, it is possible to form biofilms using only proteins. This raises the following question: what are some of the advantages of using multiple biomolecules to assemble a biofilm matrix?

A plausible explanation is that it may be beneficial for bacteria to possess redundant mechanisms of biofilm assembly, as this provides bacteria with plasticity to assemble biofilms that persist under a range of environmental conditions; having more than one way to build a biofilm better ensures that the biofilm gets built. Additionally, a proteinaceous matrix is particularly well suited to allowing the bacteria to readily remodel and disassemble via the production of proteases (20, 21, 43). Such remodeling may be essential under changing environmental factors such as competition with other bacteria, attack by host defenses, changes in flow, or altered nutrient availability (40, 44). In this way, being able to form a biofilm matrix with a unique composition as well as the ability to adapt in response to external changes may improve bacterial survival.

Most studies of P. aeruginosa biofilms have indicated a critical role for EPS in the matrix (45). Consistent with this, when we surveyed an extensive database of genomes of P. aeruginosa strains, we did not identify any strains that were missing genes for all types of EPS (Psl, Pel, and alginate) (46). This raises the following question: why produce EPS as part of the matrix when only a protein is needed? We hypothesized that the utility of CdrA in biofilms may require that its proteolytic degradation be prevented or minimized and that interaction of CdrA with EPS may protect against proteolysis of CdrA. Indeed, PK treatment of CdrA-dominant aggregates and static biofilms resulted in disassembly. Under specific circumstances, proteolytic matrix degradation may be desirable. However, a hallmark of robust biofilm formation that is associated with bacterial persistence is the presence of a matrix that is recalcitrant to damage by host molecules and other environmental assaults. Bacteria in biofilms encounter proteases from their environment (e.g., protease-rich sputum [38]) and self-produced proteases (36, 37) and therefore require a mechanism of protection against digestion. For CdrA, an interaction with Psl was found to be protective against P. aeruginosa proteases.

**FIG 7:**
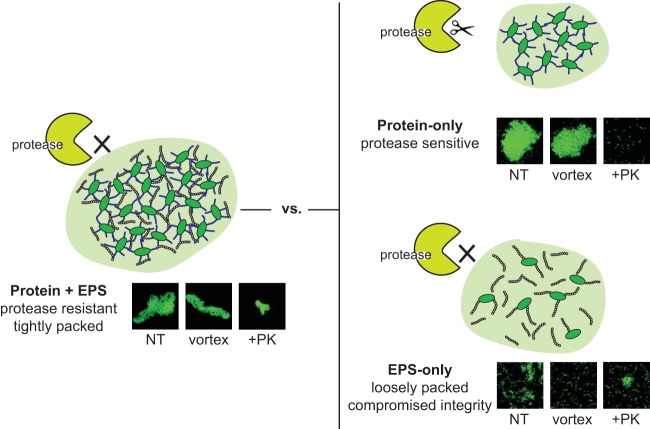
P. aeruginosa can assemble aggregates using both EPS and CdrA. When both components are part of the matrix, robust aggregates that are tightly packed and protease resistant are formed. In contrast, when only CdrA is present, the aggregates are highly susceptible to proteolytic degradation. While EPS-only aggregates are protease resistant, they assemble as loosely packed aggregates whose structural integrity is easily disrupted.

Similarly, past work has shown that P. aeruginosa biofilms that contain EPS alone are not as robust as mixed EPS-CdrA biofilms (Fig. 7) (19). While CdrA-deficient biofilms still accumulate biofilm biomass, they form loosely packed aggregates of bacteria with aberrant matrix localization and compromised integrity. In fact, aggregates of CdrA-deficient bacteria can be easily physically dislodged from the flow cell surface by altering the flow rate (19). Similar requirements for mixed EPS-protein matrices have been identified in other systems. For example, optimal biofilm formation in Vibrio cholerae requires the production of matrix proteins (RbmA, Bap1, and RbmC) and *Vibrio* polysaccharide (VPS) (47), and RbmA and VPS have been shown to interact within the matrix (48, 49). Past explanations of the necessity of multibiomolecular matrices have included studies of the improved material properties of protein-polysaccharide blends in comparison to matrices composed of only a single material (50–52).

Here we explored an additional possibility: that protein-polysaccharide blends are utilized in biofilm matrices to minimize proteolysis of CdrA and the resulting erosion of biofilm biomass that might occur in the presence of extracellular proteases. For example, using a panel of protease mutants, we determined that the P. aeruginosa elastase, LasB, can degrade CdrA unless it is protected by Psl. Expression of LasB is regulated by the quorum-sensing Las system. LasB is among the P. aeruginosa self-produced proteases that contribute to virulence by damaging the host and degrading flagella, which would otherwise elicit a host immune response (53, 54). The proteolysis of CdrA by LasB provides a potentially interesting link between quorum sensing and biofilm formation.

LasB also might provide a nonspecific mechanism for modulating bacterial aggregate growth and disassembly. Recent findings demonstrated that the c-di-GMP-regulated protease LapG can cleave CdrA at its C terminus, resulting in release of CdrA from the cellular surface under conditions of low levels of c-di-GMP (20, 21). CdrA is made under conditions of high c-di-GMP levels, creating a stable biofilm structure, and as c-di-GMP levels drop, CdrA is enzymatically cleaved from the cell surface by LapG. Under unfavorable biofilm conditions, the interaction between CdrA and Psl may be destabilized, permitting LasB to cleave CdrA and further promote disaggregation. However, when CdrA and Psl interact, the bacteria are then protected against digesting their own matrix and are still able to produce proteases that are important for virulence and/or survival. This model fits with the general finding that dispersed bacteria exhibit decreased levels of intracellular c-di-GMP and increased levels of matrix-degrading enzymes (55).

In addition to identifying an EPS-independent function for CdrA, we also showed a novel role for CdrA-Psl interactions. CdrA-Psl interactions provide structural stability via cross-linking, and we have now shown that CdrA-Psl interactions also provide protection from proteolytic degradation. This work emphasizes the importance of different biofilm matrix components and that their assembly outside the cells can provide biofilm stability.

## MATERIALS AND METHODS

### Bacterial strains and growth conditions.

Bacterial strains and plasmids used in this study are listed in Table S1 in the supplemental material. Unless otherwise noted, strains were grown at 37°C in Luria-Bertani (LB) broth.

10.1128/mBio.01376-18.9 TABLE S1 Strains, plasmids, and primers used in this study are outlined in the table. Download Table S1, DOCX file, 0.01 MB. Copyright © 2018 Reichhardt et al. 2018 Reichhardt et al. This content is distributed under the terms of the Creative Commons Attribution 4.0 International license.

### Aggregation assays.

Stationary-phase cultures were diluted 30-fold into LB medium supplemented with 1% arabinose and 300 μM carbenicillin. Mixed-culture aggregates were grown from a 1:1 inoculum of each strain. Cultures were grown in triplicate at 37°C, with shaking at 225 rpm, for 2 h 15 min. Aggregation was evaluated by visual assessment and the measurement of absorbance at 600 nm. Percent relative aggregation was calculated by taking the difference between the OD_600_ of the P*cdrAB* strain and that of its corresponding vector control strain, dividing by the OD_600_ of the vector control strain, and then multiplying by 100%. Student's *t* test was applied to determine if there was a statistically significant difference between EPS^+^ and isogenic Psl^−^ or EPS^−^ strains. For microscopy of aggregates, 20 μl of culture was deposited on a glass slide using a P200 pipette tip and imaged using confocal laser scanning microscopy with either a 20× or 63× lens objective.

For proteinase K treatment of aggregates, proteinase K (Qiagen) (final concentration, 5 mg/ml) was added to culture aliquots after 2 h 15 min of growth and incubated for 30 min at room temperature with rocking before imaging by confocal laser scanning microscopy was performed. Untreated samples were similarly incubated with rocking.

### Crystal violet assay.

Static biofilm formation was assessed using the crystal violet assay as previously described (19). Static biofilms were cultured in Nunc Bacti 96-well microtiter plates using Vogel-Bonner minimal medium (VBMM) supplemented with 0.2% arabinose and 300 μM carbenicillin. Cultures were incubated statically for 20 h at 37°C before nonadherent biomass was removed and the crystal violet assay performed. Student’s *t* test was applied to determine if there was a statistically significant difference between uninduced (“no arabinose”) and induced (“plus arabinose”) samples of the same strain background.

For proteinase K treatment of static biofilms, proteinase K (Qiagen) was added to the wells at a final concentration of 5 mg/ml after 19 h of growth, and then the reaction mixtures were statically incubated for 1 h at 37°C before the nonadherent biomass was removed and the crystal violet assay performed. Student’s *t* test was applied to determine if there was a statistically significant difference between untreated (“no treatment” [NT]) and proteinase K-treated (“PK”) samples of the same strain.

### CdrA purification.

CdrA protein expression was performed in P. aeruginosa strain MPAO1 Δ*lasR* Δ*rhlR* P*cdrAB*, and cultures were grown in LB medium supplemented with 1% arabinose and 300 μm carbenicillin. Supernatant was harvested following centrifugation of the culture for 10 min at 5,000 × *g*. Centrifugation was repeated once to remove residual cellular debris. Protease inhibitor was added to the supernatant (1 Roche tablet and 100 μl Halt protease inhibitor were added per 25-ml aliquot of supernatant). Supernatant was then concentrated using a 100-kDa Amicon filter in an Amicon stirred cell. The supernatant was treated with DNase prior to dialysis against PBS (100-kDa molecular weight cutoff [MWCO]) and then purified over a Sephacryl S-300 column. Fractions were tested for CdrA by the use of a sodium dodecyl sulfate-polyacrylamide gel electrophoresis (SDS-PAGE) gel.

### Psl isolation.

Psl was isolated from MPAO1 pBAD*psl* grown in Jensen’s medium supplemented with 2% arabinose. Cultures were grown overnight at 37°C with shaking. Cells were pelleted by centrifuging twice at 8,300 × *g* for 15 min at room temperature, and the pellet was discarded. To precipitate Psl, ice-cold ethanol was added to the supernatant at a ratio of 3:1 and the reaction mixture was incubated at 4°C for 1 h. Psl was pelleted by spinning at 8,300 × *g* for 15 min at 4°C, and the supernatant was discarded. The Psl-containing pellet was washed three times with ice-cold 95% ethanol. The pellet was then washed with 100% ice-cold ethanol, and the pellet was air dried overnight. The sample was tested for the presence of Psl by immunoblotting.

### Western blot detection.

CdrA was examined by immunoblot assays. Protein gel electrophoresis was carried out using 3 to 8% XT Tris-acetate gels (Criterion). Proteins were transferred to 0.2-μm-pore-size polyvinylidene difluoride (PDVF) transfer membranes (Bio-Rad). The primary CdrA antibody (GenScript; raised against CGDFQGRGELPRAKN) was diluted to 1/10,000 in 1% milk–Tris-buffered saline with Tween 20 (TBST). Horseradish peroxidase (HRP)-conjugated goat anti-rabbit antibody (Invitrogen) was used as the secondary antibody. Detection was performed with SuperSignal West Pico chemiluminescent substrate (Thermo Scientific).

### Protease susceptibility assay.

Purified CdrA and isolated EPS were incubated together (10 μg CdrA to 30 μg EPS) overnight at room temperature with rotation. Sterile water was added to reach a final volume of 50 μl. As a control, CdrA was incubated with sterile water alone. Cell-free supernatants from stationary-phase cultures of strain PAO1 Δ*wspF* Δ*cdrA* Δ*EPS* were added to the CdrA-polysaccharide mixtures. Two parts cell-free supernatant (or boiled supernatant or sterile water) were added to one part CdrA-polysaccharide mixture. Reaction mixtures were incubated at 37°C for 16 h or for the indicated time before immunoblot analysis was performed. The proteolysis assay of bovine serum albumin (BSA) was performed identically with the exception that BSA and Psl were incubated together at a ratio of 4 μg BSA to 30 μg Psl so that the molar equivalent of BSA was the same as for CdrA. For the assay, the Psl was isolated from P. aeruginosa. Commercially available cellulose (Sigma), chitosan (Sigma), and corn starch (Albertson’s) were used.

### Latex bead assay.

Purified protein (5 μg) was passively adsorbed onto 3-μm-diameter polystyrene latex beads (Sigma) (1% solution). Adsorption took place in 25 mM MES (morpholineethanesulfonic acid; pH 6.5) at room temperature with rotation for 48 h. Unadsorbed protein was removed by washing the beads three times with 25 mM MES (pH 6.5). The beads then were suspended in PBS and incubated for 4 h with rotation at room temperature. For imaging, the beads were diluted 5-fold in PBS and deposited onto a hanging drop slide. The slide was inverted prior to imaging so that beads were located near the cover slip.

### Zymogram gel.

Proteases secreted by stationary-phase cultures were resolved using 7.5% sodium dodecyl sulfate-polyacrylamide gel electrophoresis (SDS-PAGE) gels containing 0.2% gelatin (Sigma) and 0.2% casein (Sigma). Samples were mixed with nonreducing sample buffer and were not boiled. Following electrophoresis, the gel was washed with 2.5% Triton X-100 before incubation was performed for 48 h at 37°C in 50 mM Tris-HCl (pH 7.5)–1% Triton X-100–5 mM CaCl_2_–1 μM ZnCl_2_. The gel was then stained with Coomassie and imaged.

## References

[1] Hall-Stoodley L, Costerton JW, Stoodley P 2004 Bacterial biofilms: from the natural environment to infectious diseases. Nat Rev Microbiol 2:95–108. doi:10.1038/nrmicro821. 15040259

[2] Lebeaux D, Chauhan A, Rendueles O, Beloin C 2013 From *in vitro* to *in vivo* models of bacterial biofilm-related infections. Pathogens 2:288–356. doi:10.3390/pathogens2020288. 25437038 PMC4235718

[3] Römling U, Balsalobre C 2012 Biofilm infections, their resilience to therapy and innovative treatment strategies. J Intern Med 272:541–561. doi:10.1111/joim.12004. 23025745

[4] Costerton JW, Stewart PS, Greenberg EP 1999 Bacterial biofilms: a common cause of persistent infections. Science 284:1318–1322. doi:10.1126/science.284.5418.1318. 10334980

[5] Bjarnsholt T, Alhede M, Alhede M, Eickhardt-Sørensen SR, Moser C, Kühl M, Jensen PØ, Høiby N 2013 The *in vivo* biofilm. Trends Microbiol 21:466–474. doi:10.1016/j.tim.2013.06.002. 23827084

[6] Donlan RM, Costerton JW 2002 Biofilms: survival mechanisms of clinically relevant microorganisms. Clin Microbiol Rev 15:167–193. doi:10.1128/CMR.15.2.167-193.2002. 11932229 PMC118068

[7] Tseng BS, Zhang W, Harrison JJ, Quach TP, Song JL, Penterman J, Singh PK, Chopp DL, Packman AI, Parsek MR 2013 The extracellular matrix protects *Pseudomonas aeruginosa* biofilms by limiting the penetration of tobramycin. Environ Microbiol 15:2865–2878. doi:10.1111/1462-2920.12155. 23751003 PMC4045617

[8] Høiby N, Bjarnsholt T, Givskov M, Molin S, Ciofu O 2010 Antibiotic resistance of bacterial biofilms. Int J Antimicrob Agents 35:322–332. doi:10.1016/j.ijantimicag.2009.12.011. 20149602

[9] Doroshenko N, Tseng BS, Howlin RP, Deacon J, Wharton JA, Thurner PJ, Gilmore BF, Parsek MR, Stoodley P 2014 Extracellular DNA impedes the transport of vancomycin in *Staphylococcus epidermidis* biofilms preexposed to subinhibitory concentrations of vancomycin. Antimicrob Agents Chemother 58:7273–7282. doi:10.1128/AAC.03132-14. 25267673 PMC4249571

[10] Anderl JN, Franklin MJ, Stewart PS 2000 Role of antibiotic penetration limitation in *Klebsiella pneumoniae* biofilm resistance to ampicillin and ciprofloxacin. Antimicrob Agents Chemother 44:1818–1824. doi:10.1128/AAC.44.7.1818-1824.2000. 10858336 PMC89967

[11] Flemming HC, Wingender J 2010 The biofilm matrix. Nat Rev Microbiol 8:623–633. doi:10.1038/nrmicro2415. 20676145

[12] Høiby N, Ciofu O, Bjarnsholt T 2010 *Pseudomonas aeruginosa* biofilms in cystic fibrosis. Future Microbiol 5:1663–1674. doi:10.2217/fmb.10.125. 21133688

[13] Evans TJ 2015 Small colony variants of *Pseudomonas aeruginosa* in chronic bacterial infection of the lung in cystic fibrosis. Future Microbiol 10:231–239. doi:10.2217/fmb.14.107. 25689535

[14] Lam J, Chan R, Lam K, Costerton JW 1980 Production of mucoid microcolonies by *Pseudomonas aeruginosa* within infected lungs in cystic fibrosis. Infect Immun 28:546–556. 6772562 10.1128/iai.28.2.546-556.1980 PMC550970

[15] Serra R, Grande R, Butrico L, Rossi A, Settimio UF, Caroleo B, Amato B, Gallelli L, de Franciscis S 2015 Chronic wound infections: the role of *Pseudomonas aeruginosa* and *Staphylococcus aureus*. Expert Rev Anti Infect Ther 13:605–613. doi:10.1586/14787210.2015.1023291. 25746414

[16] Roy S, Elgharably H, Sinha M, Ganesh K, Chaney S, Mann E, Miller C, Khanna S, Bergdall VK, Powell HM, Cook CH, Gordillo GM, Wozniak DJ, Sen CK 2014 Mixed-species biofilm compromises wound healing by disrupting epidermal barrier function. J Pathol 233:331–343. doi:10.1002/path.4360. 24771509 PMC4380277

[17] Mann EE, Wozniak DJ 2012 *Pseudomonas* biofilm matrix composition and niche biology. FEMS Microbiol Rev 36:893–916. doi:10.1111/j.1574-6976.2011.00322.x. 22212072 PMC4409827

[18] Flemming HC, Neu TR, Wozniak DJ 2007 The EPS matrix: the “house of biofilm cells”. J Bacteriol 189:7945–7947. doi:10.1128/JB.00858-07. 17675377 PMC2168682

[19] Borlee BR, Goldman AD, Murakami K, Samudrala R, Wozniak DJ, Parsek MR 2010 *Pseudomonas aeruginosa* uses a cyclic-di-GMP-regulated adhesin to reinforce the biofilm extracellular matrix. Mol Microbiol 75:827–842. doi:10.1111/j.1365-2958.2009.06991.x. 20088866 PMC2847200

[20] Cooley RB, Smith TJ, Leung W, Tierney V, Borlee BR, O'Toole GA, Sondermann H 2016 Cyclic di-GMP-regulated periplasmic proteolysis of a *Pseudomonas aeruginosa* type Vb secretion system substrate. J Bacteriol 198:66–76. doi:10.1128/JB.00369-15. 26100041 PMC4686201

[21] Rybtke M, Berthelsen J, Yang L, Høiby N, Givskov M, Tolker‐Nielsen T 2015 The LapG protein plays a role in *Pseudomonas aeruginosa* biofilm formation by controlling the presence of the CdrA adhesin on the cell surface. MicrobiologyOpen 4:917–930. doi:10.1002/mbo3.301. 26458733 PMC4694147

[22] Colvin KM, Irie Y, Tart CS, Urbano R, Whitney JC, Ryder C, Howell PL, Wozniak DJ, Parsek MR 2012 The Pel and Psl polysaccharides provide *Pseudomonas aeruginosa* structural redundancy within the biofilm matrix. Environ Microbiol 14:1913–1948. doi:10.1111/j.1462-2920.2011.02657.x. 22176658 PMC3840794

[23] Hickman JW, Harwood CS 2008 Identification of FleQ from *Pseudomonas aeruginosa* as a c-di-GMP-responsive transcription factor. Mol Microbiol 69:376–389. doi:10.1111/j.1365-2958.2008.06281.x. 18485075 PMC2612001

[24] Hickman JW, Tifrea DF, Harwood CS 2005 A chemosensory system that regulates biofilm formation through modulation of cyclic diguanylate levels. Proc Natl Acad Sci U S A 102:14422–14427. doi:10.1073/pnas.0507170102. 16186483 PMC1234902

[25] Ueda A, Wood TK 2009 Connecting quorum sensing, c-di-GMP, pel polysaccharide, and biofilm formation in *Pseudomonas aeruginosa* through tyrosine phosphatase TpbA (PA3885). PLoS Pathog 5:e1000483. doi:10.1371/journal.ppat.1000483. 19543378 PMC2691606

[26] Lee VT, Matewish JM, Kessler JL, Hyodo M, Hayakawa Y, Lory S 2007 A cyclic-di-GMP receptor required for bacterial exopolysaccharide production. Mol Microbiol 65:1474–1484. doi:10.1111/j.1365-2958.2007.05879.x. 17824927 PMC2170427

[27] Byrd MS, Sadovskaya I, Vinogradov E, Lu H, Sprinkle AB, Richardson SH, Ma L, Ralston B, Parsek MR, Anderson EM, Lam JS, Wozniak DJ 2009 Genetic and biochemical analyses of the *Pseudomonas aeruginosa* Psl exopolysaccharide reveal overlapping roles for polysaccharide synthesis enzymes in Psl and LPS production. Mol Microbiol 73:622–638. doi:10.1111/j.1365-2958.2009.06795.x. 19659934 PMC4409829

[28] Jackson KD, Starkey M, Kremer S, Parsek MR, Wozniak DJ 2004 Identification of *psl*, a locus encoding a potential exopolysaccharide that is essential for *Pseudomonas aeruginosa* PAO1 biofilm formation. J Bacteriol 186:4466–4475. doi:10.1128/JB.186.14.4466-4475.2004. 15231778 PMC438565

[29] Kocharova NA, Knirel YA, Shashkov AS, Kochetkov NK, Pier GB 1988 Structure of an extracellular cross-reactive polysaccharide from *Pseudomonas aeruginosa* immunotype 4. J Biol Chem 263:11291–11295. 3136157

[30] Schürks N, Wingender J, Flemming H-C, Mayer C 2002 Monomer composition and sequence of alginates from *Pseudomonas aeruginosa*. Int J Biol Macromol 30:105–111. doi:10.1016/S0141-8130(02)00002-8. 11911901

[31] Jennings LK, Storek KM, Ledvina HE, Coulon C, Marmont LS, Sadovskaya I, Secor PR, Tseng BS, Scian M, Filloux A, Wozniak DJ, Howell PL, Parsek MR 2015 Pel is a cationic exopolysaccharide that cross-links extracellular DNA in the *Pseudomonas aeruginosa* biofilm matrix. Proc Natl Acad Sci U S A 112:11353–11358. doi:10.1073/pnas.1503058112. 26311845 PMC4568648

[32] Serra DO, Conover MS, Arnal L, Sloan GP, Rodriguez ME, Yantorno OM, Deora R 2011 FHA-mediated cell-substrate and cell-cell adhesions are critical for *Bordetella pertussis* biofilm formation on abiotic surfaces and in the mouse nose and the trachea. PLoS One 6:e28811. doi:10.1371/journal.pone.0028811. 22216115 PMC3245231

[33] Heras B, Totsika M, Peters KM, Paxman JJ, Gee CL, Jarrot RJ, Perugini MA, Whitten AE, Schembri MA 2014 The antigen 43 structure reveals a molecular Velcro-like mechanism of autotransporter-mediated bacterial clumping. Proc Natl Acad Sci U S A 111:457–462. doi:10.1073/pnas.1311592111. 24335802 PMC3890832

[34] Sherlock O, Schembri MA, Reisner A, Klemm P 2004 Novel roles for the AIDA adhesin from diarrheagenic *Escherichia coli*: cell aggregation and biofilm formation. J Bacteriol 186:8058–8065. doi:10.1128/JB.186.23.8058-8065.2004. 15547278 PMC529095

[35] Parsek MR 2016 Controlling the connections of cells to the biofilm matrix. J Bacteriol 198:12–14. doi:10.1128/JB.00865-15. 26527642 PMC4686205

[36] Toyofuku M, Roschitzki B, Riedel K, Eberl L 2012 Identification of proteins associated with the *Pseudomonas aeruginosa* biofilm extracellular matrix. J Proteome Res 11:4906–4915. doi:10.1021/pr300395j. 22909304

[37] Nicas TI, Iglewski BH 1986 Production of elastase and other exoproducts by environmental isolates of *Pseudomonas aeruginosa*. J Clin Microbiol 23:967–969. 3086372 10.1128/jcm.23.5.967-969.1986 PMC268764

[38] Staudinger BJ, Muller JF, Halldórsson S, Boles B, Angermeyer A, Nguyen D, Rosen H, Baldursson Ó, Gottfreðsson M, Guðmundsson GH, Singh PK 2014 Conditions associated with the cystic fibrosis defect promote chronic *Pseudomonas aeruginosa* infection. Am J Respir Crit Care Med 189:812–824. doi:10.1164/rccm.201312-2142OC. 24467627 PMC4225830

[39] Jacobs MA, Alwood A, Thaipisuttikul I, Spencer D, Haugen E, Ernst S, Will O, Kaul R, Raymond C, Levy R, Chun-Rong L, Guenthner D, Bovee D, Olson MV, Manoil C 2003 Comprehensive transposon mutant library of *Pseudomonas aeruginosa*. Proc Natl Acad Sci U S A 100:14339–14344. doi:10.1073/pnas.2036282100. 14617778 PMC283593

[40] Zapotoczna M, O'Neill E, O'Gara JP 2016 Untangling the diverse and redundant mechanisms of *Staphylococcus aureus* biofilm formation. PLoS Pathog 12:e1005671. doi:10.1371/journal.ppat.1005671. 27442433 PMC4956047

[41] Chapman MR, Robinson LS, Pinkner JS, Roth R, Heuser J, Hammar M, Normark S, Hultgren SJ 2002 Role of *Escherichia coli* curli operons in directing amyloid fiber formation. Science 295:851–855. doi:10.1126/science.1067484. 11823641 PMC2838482

[42] McCrate OA, Zhou X, Reichhardt C, Cegelski L 2013 Sum of the parts: composition and architecture of the bacterial extracellular matrix. J Mol Biol 425:4286–4294. doi:10.1016/j.jmb.2013.06.022. 23827139 PMC3812305

[43] Boyd CD, Smith TJ, El-Kirat-Chatel S, Newell PD, Dufrene YF, O'Toole GA 2014 Structural features of the *Pseudomonas fluorescens* biofilm adhesin LapA required for LapG-dependent cleavage, biofilm formation, and cell surface localization. J Bacteriol 196:2775–2788. doi:10.1128/JB.01629-14. 24837291 PMC4135675

[44] Bridier A, Piard J-C, Pandin C, Labarthe S, Dubois-Brissonnet F, Briandet R 2017 Spatial organization plasticity as an adaptive driver of surface microbial communities. Front Microbiol 8:1364. doi:10.3389/fmicb.2017.01364. 28775718 PMC5517491

[45] Colvin KM, Gordon VD, Murakami K, Borlee BR, Wozniak DJ, Wong GC, Parsek MR 2011 The Pel polysaccharide can serve a structural and protective role in the biofilm matrix of *Pseudomonas aeruginosa*. PLoS Pathog 7:e1001264. doi:10.1371/journal.ppat.1001264. 21298031 PMC3029257

[46] Brittnacher MJ, Fong C, Hayden HS, Jacobs MA, Radey M, Rohmer L 2011 PGAT: a multistrain analysis resource for microbial genomes. Bioinformatics 27:2429–2430. doi:10.1093/bioinformatics/btr418. 21765097 PMC3157930

[47] Berk V, Fong JCN, Dempsey GT, Develioglu ON, Zhuang X, Liphardt J, Yildiz FH, Chu S 2012 Molecular architecture and assembly principles of *Vibrio cholerae* biofilms. Science 337:236–239. doi:10.1126/science.1222981. 22798614 PMC3513368

[48] Smith DR, Maestre-Reyna M, Lee G, Gerard H, Wang AH-J, Watnick PI 2015 In situ proteolysis of the *Vibrio cholerae* matrix protein RbmA promotes biofilm recruitment. Proc Natl Acad Sci U S A 112:10491–10496. doi:10.1073/pnas.1512424112. 26240338 PMC4547210

[49] Fong JCN, Rogers A, Michael AK, Parsley NC, Cornell WC, Lin Y-C, Singh PK, Hartmann R, Drescher K, Vinogradov E, Dietrich LEP, Partch CL, Yildiz FH 2017 Structural dynamics of RbmA governs plasticity of *Vibrio cholerae* biofilms. Elife 6:e26263. doi:10.7554/eLife.26163. PMC5605196 28762945

[50] Hollenbeck EC, Fong JCN, Lim JY, Yildiz FH, Fuller GG, Cegelski L 2014 Molecular determinants of mechanical properties of *V. cholerae* biofilms at the air-liquid interface. Biophys J 107:2245–2252. doi:10.1016/j.bpj.2014.10.015. 25418293 PMC4241461

[51] Saldaña Z, Xicohtencatl-Cortes J, Avelino F, Phillips AD, Kaper JB, Puente JL, Girón JA 2009 Synergistic role of curli and cellulose in cell adherence and biofilm formation of attaching and effacing *Escherichia coli* and identification of Fis as a negative regulator of curli. Environ Microbiol 11:992–1006. doi:10.1111/j.1462-2920.2008.01824.x. 19187284 PMC2672964

[52] Zhang R, Xia A, Ni L, Li F, Jin Z, Yang S, Jin F 2017 Strong shear flow persister bacteria resist mechanical washings on the surfaces of various polymer materials. Adv Biosys 1:1700161. doi:10.1002/adbi.201700161. 32646157

[53] Preston MJ, Seed PC, Toder DS, Iglewski BH, Ohman DE, Gustin JK, Goldberg JB, Pier GB 1997 Contribution of proteases and LasR to the virulence of Pseudomonas aeruginosa during corneal infections. Infect Immun 65:3086–3090. 9234758 10.1128/iai.65.8.3086-3090.1997 PMC175435

[54] Casilag F, Lorenz A, Krueger J, Klawonn F, Weiss S, Haussler S 2016 The LasB elastase of *Pseudomonas aeruginosa* acts in concert with alkaline protease AprA to prevent flagellin-mediated immune recognition. Infect Immun 84:162–171. doi:10.1128/IAI.00939-15. 26502908 PMC4693985

[55] Petrova OE, Sauer K 2016 Escaping the biofilm in more than one way: desorption, detachment or dispersion. Curr Opin Microbiol 30:67–78. doi:10.1016/j.mib.2016.01.004. 26826978 PMC4821722

