# Supplementary material for: CdrA Interactions within the Pseudomonas aeruginosa Biofilm Matrix Safeguard It from Proteolysis and Promote Cellular Packing
Source: mBio. 2018 Sep 25;9(5):e01376-18. doi: 10.1128/mBio.01376-18 (PMC6156197; doi:10.1128/mBio.01376-18)
Supplement: TABLE S1 [file mbo004184067st1.docx]

**Supplementary Table 1.** Strains, plasmids, and primers

| ***P. aeruginosa* strains** | | **Reference** |
| --- | --- | --- |
| PAO1 | Wild type | Holloway, 1955 |
| PAO1 Δ*cdrA* | *cdrA,* nonpolar mutation | This study |
| PAO1 Δ*cdrA/psl* | *cdrA,* nonpolar mutation; *pslBCD* polar mutant of *psl* operon | Colvin, 2013; This study |
| PAO1 Δ*cdrA/EPS* | *cdrA,* nonpolar mutation; *pslBCD,* polar mutant of *psl* operon; *pelA,* polar mutant of the *pel* operon; *algD* nonpolar mutation | Colvin, 2013; This study |
| PAO1 Δ*wspF* | *wspF*, nonpolar mutation | Borlee, 2010 |
| PAO1 Δ*wspF/cdrA* | *wspF*, nonpolar mutation*; cdrA,* nonpolar mutation | Borlee, 2010 |
| PAO1 Δ*wspF/cdrA/psl* | *wspF*, nonpolar mutation*; cdrA,* nonpolar mutation; *pslBCD* polar mutant of *psl* operon | Colvin, 2013; This study |
| PAO1 Δ*wspF/cdrA/EPS* | *wspF*, nonpolar mutation*; cdrA,* nonpolar mutation; *pslBCD,* polar mutant of *psl* operon; *pelA,* polar mutant of the *pel* operon; *algD* nonpolar mutation | Colvin, 2013; This study |
| ***P. aeruginosa* protease mutants** | |  |
| Aminopeptidase-1 | PW5943, phoAbp01q2A11, PA2939 | Jacobs, 2003 |
| Aminopeptidase-2 | PW5944, phoAbp01q4F08, PA2939 | Jacobs, 2003 |
| AprA-1 | PW3252, lacZwp01q1E10, PA1249 | Jacobs, 2003 |
| AprA-2 | PW3253, lacZwp06q4A06, PA1249 | Jacobs, 2003 |
| Protease IV-1 | PW8078, phoAbp01q4C08, PA4175 | Jacobs, 2003 |
| Protease IV-2 | PW8077, phoAbp01q3G10, PA4175 | Jacobs, 2003 |
| PasP-1 | PW1774, lacZwp09q1C07, PA0423 | Jacobs, 2003 |
| PasP-2 | PW1775, phoAbp01q4G10, PA0423 | Jacobs, 2003 |
| LasA-1 | PW4282, lacZwp08q1H03, PA1871 | Jacobs, 2003 |
| LasA-2 | PW4283, phoAbp02q1H11, PA1871 | Jacobs, 2003 |
| LasB-1 | PW7303, phoAbp03q2F06, PA3724 | Jacobs, 2003 |
| LasB-2 | PW7302, phoAbp02q4F10, PA3724 | Jacobs, 2003 |

| ***E. coli*** | |  |
| --- | --- | --- |
| DH5α p-Δ*cdrA­* | cloning strain carrying plasmid with *cdrA* deletion allele | This Study |
| **Primers** | |  |
| cdrAUpF01 | GGGGACAAGTTTGTACAAAAAAGCAGGCTCAATCGGCACCTTGTTGCTGATCG |  |
| cdrAUpR01 | ATAGTGCGCATTGCGAAAAGGGGAAAATCTCCCTATCTGCGTGGCGC |  |
| cdrADownF02 | CCCTTTTCGCAATGCGCACTAT |  |
| cdrADownR02 | GGGGACCACTTTGTACAAGAAAGCTGGGTATCGGCCAGGGACACCTGCTC |  |
| cdrAUPF-SEQ | GGAGGCATGGTCGAGGAAAA |  |
| cdrADOWNR-SEQ | GTACAGTCCCTGGCAACTCC |  |
